# Feasibility of Multiple Repeat Gamma Knife Radiosurgeries for Trigeminal Neuralgia: A Case Report and Review of the Literature

**DOI:** 10.1155/2011/258910

**Published:** 2011-09-04

**Authors:** Guy C. Jones, Ameer L. Elaimy, John J. Demakas, Hansi Jiang, Wayne T. Lamoreaux, Robert K. Fairbanks, Alexander R. Mackay, Barton S. Cooke, Christopher M. Lee

**Affiliations:** ^1^Department of Radiation Oncology, Gamma Knife of Spokane, 910 West 5th Avenue, Suite 102, Spokane, WA 99204, USA; ^2^Department of Radiation Oncology, Cancer Care Northwest, 910 West 5th Avenue, Suite 102, Spokane, WA 99204, USA; ^3^Department of Medical Education, Providence Sacred Heart Medical Center, 101 West 8th Avenue, Spokane, WA 99204, USA; ^4^Department of Natural Sciences, Carroll College, 1601 North Benton Avenue, Helena, MT 59625, USA; ^5^Department of Neurosurgery, Spokane Brain & Spine, 801 West 5th Avenue, Suite 210, Spokane, WA 99204, USA; ^6^Department of Medical Education, University of Washington School of Medicine, 325 9th Avenue, Seattle, WA 98104, USA; ^7^Department of Neurosurgery, MacKay & Meyer MDs, 711 S Cowley Street, Suite 210, Spokane, WA 99202, USA

## Abstract

Treatment options for trigeminal neuralgia (TN) must be customized for the individual patient, and physicians must be aware of the medical, surgical, and radiation treatment modalities to prescribe optimal treatment courses for specific patients. The following case illustrates the potential for gamma knife radiosurgery (GKRS) to be repeated multiple times for the purpose of achieving facial pain control in cases of TN that have been refractory to other medical and surgical options, as well as prior GKRS. The patient described failed to achieve pain control with initial GKRS, as well as medical and surgical treatments, but experienced significant pain relief for a period of time with a second GKRS procedure and later underwent a third procedure. Only a small subset of patients have reportedly undergone more than two GKRS for TN; thus, further research and long-term clinical followup will be valuable in determining its usefulness in specific clinical situations.

## 1. Introduction

Trigeminal neuralgia (TN), also known as *tic douloureux*, is a disorder of the sensory nucleus of cranial nerve V, which causes severe episodic shooting pains in one or more of its three divisions (V1–V3). TN is most commonly idiopathic, but may be caused by pressure from a structure, such as a blood vessel compressing or pulsating on the trigeminal nerve or its vasculature. This condition affects females twice as often as males, with a peak incidence at 60 years of age [1]. Triggers for episodes of pain vary greatly among individuals, with patients commonly reporting pain with brushing teeth, chewing, talking, touching the face, and cold sensations on the face or teeth. Options for management of TN include medical, surgical, and radiation approaches. This paper describes a rare and unique course of treatment for TN due to the refractory nature of the disease process and because the patient received three separate gamma knife radiosurgery (GKRS) treatments. This course of treatment may prove useful in a selected group of patients with a similar clinical situation.

## 2. Case Report

A 72-year-old man was referred to a community oncology center by his neurologist due to complaints of a recurrence of lancinating, “shock-like” pain in the left side of his face from the preauricular area into the left side of his upper and lower jaws. The patient stated that the pain was intermittent, 10/10 in intensity (on a scale of 1 to 10, with 10 being the worst), and was triggered by several actions including eating, brushing gums, cold sensations, and touching his face. The patient stated that the pain was accompanied by tenderness and sensitivity to touch around the outside margin of his left eye, but he denied numbness or other change in sensation in the affected area. 

The patient stated that his symptoms began as an “annoyance” approximately 4.5 years prior and progressed slowly over the course of the next 3 years, at which time he was diagnosed with TN by his dentist. An MRI of the brain performed at the time of diagnosis demonstrated a tortuous basilar artery abutting the left trigeminal nerve. He was initially started on Gabapentin, which provided only mild relief. Carbamazepine was added later, but it caused him to become excessively drowsy, which resulted in a significant fall and concussion. Due to his fall, the medications were discontinued. 

**Figure 1:**
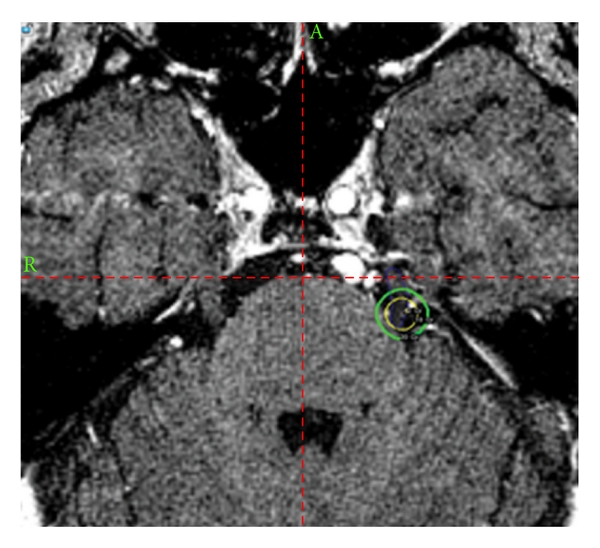
Axial section through the brainstem at the nerve root entry zone of the left trigeminal nerve with an illustration of the location of the 50% isodose line for gamma knife radiation treatment planning. This was the treatment location for the first and second gamma knife procedures.

Following failure of medical management, the patient was offered further treatment options including microvascular decompression (MVD), radiofrequency gangliotomy, and GKRS. The patient elected to undergo GKRS which was performed with a Leksell model C gamma knife to the trigeminal nerve root entry zone with 201 cobalt sources (4 mm shot size) to a dose of 42 Gy prescribed to the 50% isodose line for a maximum point dose of 84 Gy (see Figure 1). Shortly after the procedure, the patient experienced mild relief of pain but had a sudden recurrence in the second division of the trigeminal nerve within 2 months. In hopes of achieving further pain relief, the patient underwent a radiofrequency gangliotomy procedure at a major university medical center 7 months after his GKRS, which provided pain relief for a period of 4 months. However, the patient's pain recurred and became debilitating leading him to again seek treatment. A second GKRS procedure was performed 1 month after pain recurrence (approximately 11 months after the first one), with the same proximal nerve root entry zone targeted to a dose of 27 Gy to the 50% isodose line for a maximum dose of 54 Gy at the center point. At that point, the patient had a maximum dose of 138 Gy to the nerve. Shortly after the procedure, the patient experienced nearly complete resolution of his pain for the next 6.5 months and was able to discontinue all oral pain medications. However, the patient began to experience gradual recurrence of the pain in his left upper and lower jaws and again sought evaluation for further pain control.

**Figure 2:**
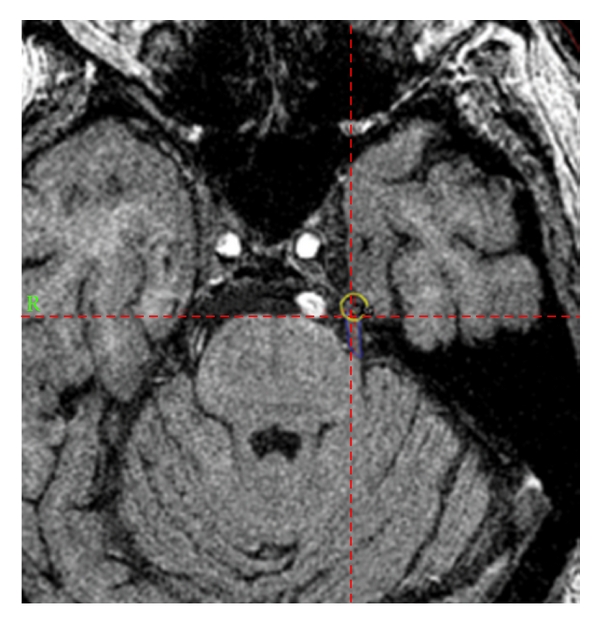
Axial section through the brainstem at the nerve root entry zone of the left trigeminal nerve with an illustration of the location of the 50% isodose line for the gamma knife radiation treatment planning. This was the location of treatment for his third course of treatment.

Medical, surgical, and radiosurgical pain management options were again discussed with the patient. He stated that he would prefer to avoid surgery and had difficulty tolerating pain medications, which he reported made him feel drowsy and disoriented. The patient stated that he would prefer to have a 3rd GKRS procedure because it was the only treatment that had provided him extended and significant pain relief. The patient underwent his 3rd GK treatment approximately 17 months after his first one. The 3rd treatment was delivered a few millimeters distal from the previous target and was prescribed 20 Gy (with 4 mm shots) to the 50% isodose line (see Figure 2) for a maximum point dose of 40 Gy. This lower treatment dose and more distal target were chosen in an attempt to limit neural toxicity from the already high cumulative dose to the nerve. The 50% isodose line for the third target was set on the nerve to match the 50% isodose line location for the previous two targets. Therefore, there was some additional dose fall-off into the previously treated region from the lower isodose regions.

The patient was symptom free and reported no side effects or focal neurological problems for 3 months after the procedure. At that point, he experienced recurrence of his pain and underwent a microvascular decompression (MVD). At his 6 month followup from MVD, he reported having no pain or facial numbness. 

Since initial presentation, the patient has had regular follow-up visits with his primary care physician, neurosurgeon, and radiation oncologist. The patient's reported lack of facial numbness and other side effects were verified through follow-up physical examinations. He will continue to be followed closely by his treating physicians in the future and understands that with retreatment his risks of permanent side effects are increased.

## 3. Discussion

Optimal treatment of TN remains challenging, as each clinical situation can vary significantly. This case illustrates a unique approach to the management of TN in that this patient has received 3 separate GKRS treatments to the same nerve root for refractory disease. Patients who suffer from TN have a number of treatment modalities to consider, and treatments should be tailored to the individual situation. 

Medications in the form of anticonvulsants, such as Gabapentin and Carbamazepine, as well as antidepressants, are the predominant method for treating TN-related facial pain. However, there is a fraction of patients who experience only limited relief from pharmacotherapy or are unable to endure the side effects of the prescribed drugs and, thus, seek other treatment alternatives [2]. 

Neurosurgical intervention is often the next line of treatment for patients where pharmaceuticals have failed. Microvascular decompression (MVD) is a procedure that involves a craniotomy to locate and separate veins or arteries in contact with the trigeminal nerve, while preserving its function [3]. MVD has been proven to provide patients with TN pain relief, but carries with it the risks of neurosurgical complications which may not be acceptable for patients with certain comorbidities [4]. Percutaneous rhizotomies are another set of neurosurgical procedures that create a permanent lesion at the trigeminal root or ganglion by thermal, chemical, or mechanical means [3]. These procedures are generally regarded as safe and effective with low incidence of unwanted side effects [5] such as nerve damage [3, 4] and vascular injury. 

 GKRS has been shown to be safe and effective in patients with medically [6] and surgically [7] refractive TN. On an increasing number of occasions, GKRS is attempted a second time and in only a handful of cases have outcomes been reported in the literature where a 3rd treatment was performed, making it largely uncharted territory. Repeat GKRS, in cases where it has previously been effective, have reported similar rates of complete pain control as with the initial procedure [8]. However, successful retreatment of patients in whom the initial GK treatment fails is also feasible [9], as was illustrated in this case with the second GKRS. Overall, repeat GKRS has been shown to provide significant pain relief in more than 2/3 of patients [10, 11], with some studies showing similar [12, 13] and others showing better [11] overall facial-pain outcomes than primary radiosurgery. Studies have also demonstrated that lack of a prior neurosurgical procedure was predictive of better pain control [14, 15].

Pollock and Stein investigated the effect of different treatment modalities for idiopathic TN in patients who had undergone three or more previous operations of any kind. This study found that posterior fossa exploration resulted in better facial pain outcomes than SRS or percutaneous techniques in this group of patients; however, this paper acknowledges selection bias in their choice of treatment [16]. Based on the fact that the patient in this paper had at least 3 interventions for pain relief prior to his 3rd GKRS, the Pollock study would have predicted that he had a 36% chance of complete pain relief after 3 years and a 45% chance of new facial numbness or dysesthetic pain. It should be noted that the study did not mention whether any patient underwent 3 GKRS making it difficult to stratify the likely outcome. Another study by Gellner et al. reported two patients, each had four GKRS operations, but gave few details about those specific cases or their outcomes [10].

Studies disagree about the incidence of complications in primary versus repeat GKRS, with some showing no significant increase in incidence of complications beyond that observed in the initial procedure [8], and others showing a significant difference for side effects, such as facial numbness [9, 11, 13, 17, 18]. Huang et al. suggested that the incidence of facial numbness was significantly increased above a cumulative dose of 115 Gy [17, 19]. Dvorak et al. showed a dose-response relationship for both pain control and development of side effects, with doses above 130 Gy more likely to result in a new dysfunction, as well as improved pain control [9]. Other studies have demonstrated the same correlation between pain control and development of unwanted side effects [18–20]. Another study notes that at cumulative doses above 163 Gy, the rate of bothersome numbness was in the range of 16% [11]. In the presented case, an attempt was made to reduce neural toxicity by lowering the 2nd and 3rd GKRS doses to maximum-point doses of 54 and 40 Gy, respectively. It was felt that repeating the initial dose of 42 Gy to the 50% isodose line (84 Gy maximum) would too greatly increase the patient's likelihood of bothersome side effects. 

The radiosurgery target area may also play a significant role in both maximizing pain control and limiting side effects. Zhang et al. showed that increasing the isocenter distance between the two radiosurgeries was associated with improved pain relief, regardless of whether the second isocenter was placed proximal or distal to the first [21]. Although this study found no relationship between distance between isocenters (in first and second radiosurgeries) and occurrence of dysesthesias, only five patients with dysesthesias were included in this segment of the study. Using this premise, the treatment isocenter for the 3rd GKRS was moved distally in hopes of achieving a better clinical response while limiting side effects.

## 4. Conclusion

This paper highlights a case of TN refractory to initial medical and surgical management, nonresponsive to an initial GKRS procedure, responsive to second and third GKRS procedures, and having undergone an MVD due to refractory pain after a pain-free interval. This is one of very few reported cases of a patient undergoing three GKRS procedures for TN. Further research will be valuable in determining its usefulness in clinical practice.
